# Exploring the causal association between frailty index with the common types of arthritis: a Mendelian randomization analysis

**DOI:** 10.1007/s40520-024-02813-8

**Published:** 2024-08-12

**Authors:** Weichu Sun, Hui Xiao, Yayun Li

**Affiliations:** 1 Department of Health Management, The Third Xiangya Hospital, Central South University, Changsha, China; 2 Department of Orthopaedics, Xiangya Hospital, Central South University, Changsha, China; 3 Department of Dermatology, Xiangya Hospital, Central South University, Changsha, China; 4 Hunan Engineering Research Center of Skin Health and Disease, Xiangya Hospital, Central South University, Changsha, China; 5 Hunan Key Laboratory of Skin Cancer and Psoriasis, Xiangya Hospital, Central South University, Changsha, China; 6 National Clinical Research Center for Geriatric Disorders, Xiangya Hospital, Central South University, Changsha, China

**Keywords:** Mendelian randomization study, Arthritis, Frailty, Older people

## Abstract

**Background:**

Previous observational studies indicated a complex association between frailty and arthritis.

**Aims:**

To investigate the genetic causal relationship between the frailty index and the risk of common arthritis.

**Methods:**

We performed a large-scale Mendelian randomization (MR) analysis to assess frailty index associations with the risk of common arthritis in the UK Biobank (UKB), and the FinnGen Biobank. Summary genome-wide association statistics for frailty, as defined by the frailty index, and common arthritis including rheumatoid arthritis (RA), osteoarthritis (OA), psoriatic arthritis (PSA), and ankylosing spondylitis (AS). The inverse-variance weight (IVW) method served as the primary MR analysis. Heterogeneity testing and sensitivity analysis were also conducted.

**Results:**

Our results denoted a genetic association between the frailty index with an increased risk of OA, the odds ratio (OR)_IVW_ in the UKB was 1.03 (95% confidence interval [CI]: 1.01–1.05; *P* = 0.007), and OR_IVW_ was 1.55 (95% CI: 1.16–2.07; *P* = 0.003) in the FinnGen. For RA, the OR_IVW_ from UKB and FinnGen were 1.03 (1.01–1.05, *P* = 0.006) and 4.57 (1.35–96.49; *P* = 0.025) respectively. For PSA, the frailty index was associated with PSA (OR_IVW_ = 4.22 (1.21–14.67), *P* = 0.023) in FinnGen, not in UKB (*P* > 0.05). However, no association was found between frailty index and AS (*P* > 0.05). These results remained consistent across sensitivity assessments.

**Conclusion:**

This study demonstrated a potential causal relationship that genetic predisposition to frailty index was associated with the risk of arthritis, especially RA, OA, and PSA, not but AS. Our findings enrich the existing body of knowledge on the subject matter.

**Supplementary Information:**

The online version contains supplementary material available at 10.1007/s40520-024-02813-8.

## Introduction

Arthritis is a common joint disorder characterized by joint pain, joint stiffness, or joint deformity, leading to significant impacts on patients’ quality of life, daily activities, and disease burden [1, 2]. The most common types of arthritis include osteoarthritis (OA), rheumatoid arthritis (RA), psoriatic arthritis (PsA), and ankylosing spondylitis (AS) [3, 4]. The prevalence of arthritis has recently escalated, rendering it a major global health concern. Previous studies have indicated that the aforementioned most common types of arthritis are closely associated with genetic, environmental, and age factors. However, the pathogenesis of arthritis is complex and remains to be explained.

Frailty is a complex clinical syndrome and it presents as a decline in functioning across multiple physiological systems and a reduced ability to maintain homeostasis in response to stressors [5–7]. The frailty index (FI) is a reliable tool for evaluating frailty, calculated by counting the number of health deficits throughout the individual’s life, a higher frailty index score showed significant predictive capability for various adverse health outcomes [8]. Extensive research has indicated a close association between frailty and multiple adverse conditions, including falls and fractures, deteriorating mobility, disability, and even mortality [9–11]. Additionally, the associations between frailty and arthritis are particularly prominent. Previous epidemiological research investigated the relationship between frailty and common types of arthritis. For example, lots of observational and meta-analysis studies demonstrated that RA, OA, AS, and PSA were associated with the risk of frailty [12–16]. Only one cohort study reported the association between frailty and the risk of RA, and it suggested that frailty may be an important risk factor for the deterioration of physical function over time in individuals with RA [17].

Although there is limited availability of related observational data on the association of frailty with other common types of arthritis, the epidemiological evidence linking frailty to the risk of common types of arthritis is primarily derived from observational studies. However, these studies often face challenges in avoiding the influence of confounding factors and reverse causation bias, which hinders a comprehensive understanding of the exact causality between frailty and common types of arthritis.

To address these limitations, Mendelian randomization (MR) is an alternative approach to assess causal questions by utilizing genetic variation as an instrumental variable (IVs) [18]. This approach effectively reduces the influence of confounding factors and minimizes reverse causality bias by utilizing alleles that are randomly distributed and unaffected by the occurrence of the disease [19]. Currently, MR analysis has been widely utilized to explore causal associations between exposure factors and skeletal diseases [20–23]. In this study, we aim to investigate the causal association between the frailty index and common types of arthritis (OA, RA, PSA, and AS) by employing MR analysis.

## Method

### Study design

**Fig. 1:**
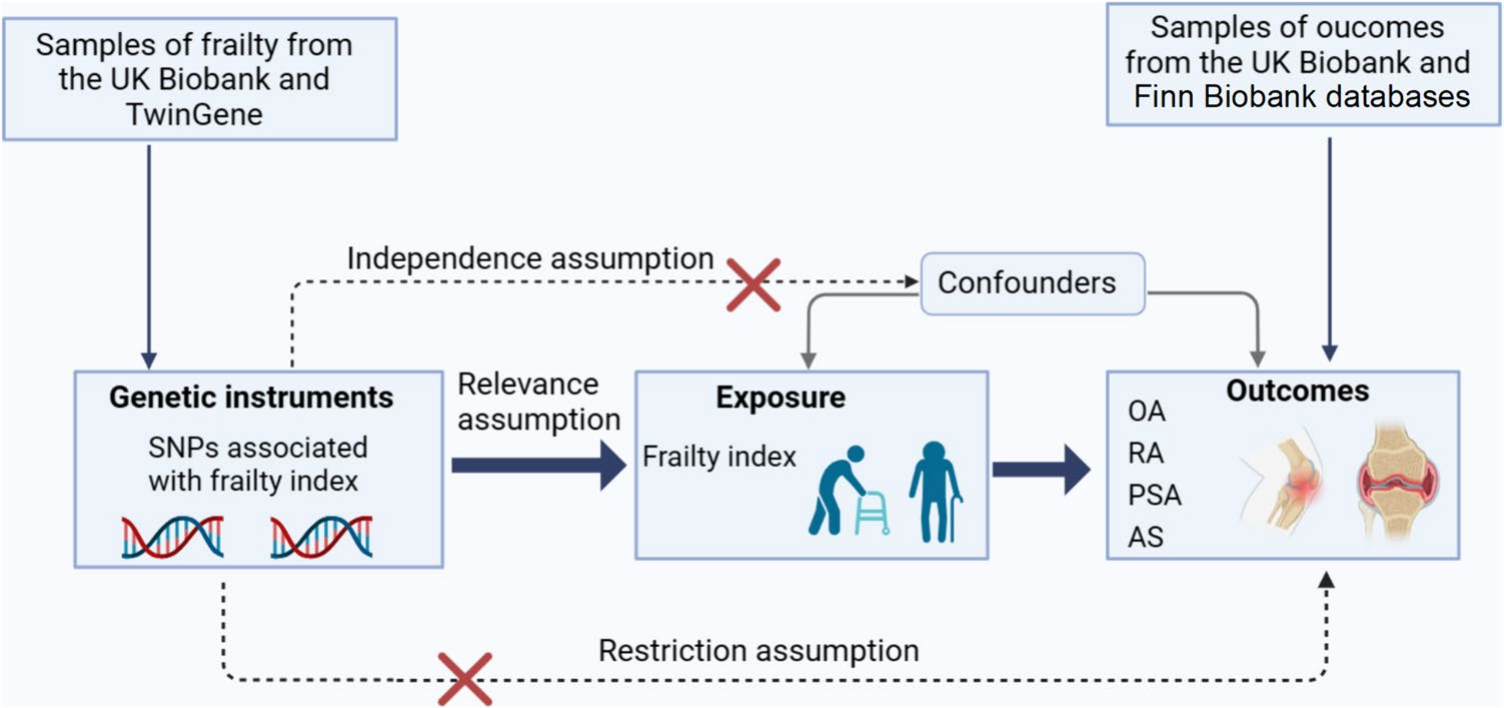
Diagram for key assumptions of Mendelian randomization analyses

This study was designed as a two-sample MR research, and the general design was illustrated in Fig. 1. To investigate the causal associations between frailty index and common types of arthritis, it is important to select valid instrumental variables (IVs) that fulfill the following three key assumptions [24]: (1) IVs are associated with exposure (frailty index), (2) IVs must be independent of any confounders, and (3) IVs can only affect the outcomes (OA, RA, PSA, and AS) through exposure. The MR analysis was used to evaluate the potential causal relationship between frailty index and types of arthritis.

### Data source

We screened datasets of the Genome-Wide Association Studies (GWAS) data about frailty index and common types of arthritis were obtained from the IEU open database (https://gwas.mrcieu.ac.uk/), Neale Lab consortium and the FinnGen database (version R10, https://www.finngen.fi/en) (Table 1). All data in this study were obtained from public databases, and ethical approvals were gained in all original papers, thereby no ethical approval and informed consent were required.

**Table 1:** The casual effects of frailty index on common types of arthritis estimated by Mendelian randomization

Exposure	Outcome	Source	nSNP	MR-method	Beta (SE)	OR (95% CI)	*P* value	Adjusted OR (95% CI)	Adjusted *P* value
Frailty index	Osteoarthritis	UKB	15	Inverse variance weighted	0.03 (0.01)	1.03 (1.01, 1.05)	0.007	1.03 (1.01, 1.05)	0.001
				Weighted median	0.02 (0.01)	1.02 (1.00, 1.04)	0.107	1.02 (1.00, 1.04)	0.081
				MR Egger	−0.08 (0.04)	0.92 (0.85, 1.00)	0.066	0.97 (0.85, 1.11)	0.656
				Simple mode	0.01 (0.02)	1.01 (0.97, 1.05)	0.553	1.01 (0.97, 1.06)	0.530
				Weighted mode	0.00 (0.01)	1.00 (0.98, 1.03)	0.851	1.01 (0.98, 1.05)	0.512
		FINN	14	Inverse variance weighted	0.44 (0.15)	1.55 (1.16, 2.07)	0.003	NA	NA
				Weighted median	0.25 (0.13)	1.29 (1.00, 1.65)	0.047	NA	NA
				MR Egger	−0.17 (0.68)	0.84 (0.22, 3.18)	0.807	NA	NA
				Simple mode	0.27 (0.20)	1.31 (0.89, 1.93)	0.196	NA	NA
				Weighted mode	0.21 (0.14)	1.24 (0.93, 1.64)	0.165	NA	NA
Frailty index	Rheumatoid arthritis	UKB	15	Inverse variance weighted	0.03 (0.01)	1.03 (1.01, 1.05)	0.006	1.01 (1.00, 1.02)	4.61E−04
				Weighted median	0.01 (0.00)	1.01 (1.00, 1.02)	0.027	1.01 (1.00, 1.02)	0.029
				MR Egger	0.18 (0.01)	1.20 (1.15, 1.24)	2.08E−07	1.07 (1.02, 1.13)	0.016
				Simple mode	0.01 (0.01)	1.01 (0.99, 1.02)	0.403	1.01 (0.99, 1.22)	0.426
				Weighted mode	0.01 (0.01)	1.01 (0.99, 1.02)	0.389	1.01 (0.99, 1.22)	0.413
		FINN	14	Inverse variance weighted	2.43 (1.09)	4.57 (1.35, 96.49)	0.025	2.29 (1.26, 4.18)	0.007
				Weighted median	0.74 (0.38)	2.09 (1.0, 4.38)	0.051	2.12 (0.96, 4.68)	0.064
				MR Egger	18.46 (2.13)	1.04E + 08 (1.60E + 06, 6.71E + 09)	1.62E−06	8.91 (0.03, 3088.28)	0.482
				Simple mode	0.72 (0.60)	2.05 (0.63, 6.66)	0.255	1.96 (0.58, 6.57)	0.302
				Weighted mode	0.72 (0.58)	2.05 (0.65, 6.44)	0.242	2 (0.60, 6.64)	0.286
Frailty index	Psoriatic arthropathies	UKB	14	Inverse variance weighted	0.66 (0.57)	1.94 (0.64, 5.89)	0.242	1.00 (0.54, 1.83)	0.994
				Weighted median	0.10 (0.38)	1.10 (0.53, 2.31)	0.793	1.07 (0.48, 2.36)	0.870
				MR Egger	7.71 (1.54)	2224.10 (109.55, 45,153.86)	3.00E−04	0.61 (0.00, 209.92)	0.871
				Simple mode	−0.35 (0.67)	0.70 (0.19, 2.60)	0.608	0.54 (0.16, 1.86)	0.351
				Weighted mode	−0.26 (0.65)	0.77 (0.22, 2.78)	0.702	0.54 (0.15, 2.03)	0.385
		FINN	14	Inverse variance weighted	1.44 (0.64)	4.22 (1.21, 14.67)	0.023	NA	NA
				Weighted median	2.33 (0.68)	10.23 (2.68, 39.07)	0.001	NA	NA
				MR Egger	7.38 (2.42)	1609.46 (13.94, 185,787.49)	0.010	NA	NA
				Simple mode	2.44 (1.19)	11.49 (1.11, 118.81)	0.061	NA	NA
				Weighted mode	2.60 (1.01)	13.46 (1.88, 96.58)	0.023	NA	NA
Frailty index	Ankylosing spondylitis	UKB	12	Inverse variance weighted	0.00 (0.00)	1.00 (0.99, 1.01)	0.763	1.00 (0.99, 1.00)	0.063
				Weighted median	0.00 (0.00)	1.00 (0.99, 1.00)	0.749	1.00 (0.99, 1.00)	0.368
				MR Egger	0.02 (0.01)	1.02 (1.01, 1.04)	0.019	0.98 (0.95, 1.01)	0.153
				Simple mode	0.00 (0.00)	1.00 (0.99, 1.01)	0.680	1.00 (0.99, 1.01)	0.732
				Weighted mode	0.00 (0.01)	1.00 (0.99, 1.01)	0.778	1.00 (0.99, 1.01)	0.769
		FINN	14	Inverse variance weighted	0.52 (0.51)	1.67 (0.62, 4.55)	0.313	NA	NA
				Weighted median	0.69 (0.70)	1.99 (0.50, 7.90)	0.329	NA	NA
				MR Egger	2.40 (2.35)	11.02 (0.11, 1094.65)	0.327	NA	NA
				Simple mode	0.00 (1.25)	1.00 (0.09, 11.45)	0.997	NA	NA
				Weighted mode	0.68 (1.00)	1.97 (0.28, 13.96)	0.509	NA	NA

We first did a raw MR analysis and got an uncorrected causal evaluation, and then, MR-PRESSO global and Outliers test was performed to find unstable SNPs (The *P* value of SNP < 1), and an adjusted MR analysis was performed again after removing them, and the causal effect values were re-evaluated

*FINN* FinnGen, *IVW* inverse variance weighted, *UKB* UK Biobank

In our MR study, we considered the frailty index as the exposure, and the common types of arthritis as the outcome. The data for the frailty index was extracted from 175,226 European ancestry subjects from the UK Biobank and TwinGene, aged 41–87 [8]. Common types of arthritis in this study encompass OA, RA, PSA, and AS. For OA, one set of data was obtained from the IEU open database, which included 38,472 cases and 424,461 controls from the United Kingdom Biobank (UKB), totaling 9,851,867 SNPs [25]. Another set of data was sourced from the FinnGen Biobank, comprising 82,596 cases and 429,209 controls (https://r10.risteys.finngen.fi/endpoints/M13_ARTHROSIS). For RA, all data was obtained from the IEU open database. One dataset consisted of 5201 cases and 457,732 controls from the UK Biobank (https://gwas.mrcieu.ac.uk/datasets/ukb-b-9125/), while another dataset was from the FinnGen Biobank, which included 6236 cases and 147,221 controls (https://gwas.mrcieu.ac.uk/datasets/finn-b-M13_RHEUMA/). For PSA, one data was obtained from the IEU open database, which comprising a sample size of 1553 cases and 147,221 controls (https://gwas.mrcieu.ac.uk/datasets/finn-b-M13_PSORIARTH/), and another GWAS summary data of PSA was gained from the Neale Lab consortium. For AS, we used two GWAS summary data, one including 166,144 European ancestry individuals (1462 cases and 164,682 controls) (https://gwas.mrcieu.ac.uk/datasets/finn-b-M13_ANKYLOSPON/), and another included 1296 cases and 461,637 controls (https://gwas.mrcieu.ac.uk/datasets/ukb-b-18194/) (Supplementary Table S1).

### Instrumental variables and MR analysis

We extracted single-nucleotide polymorphisms (SNPs) as IVs up to the genome-wide significance threshold (*P* < 5 × 10^−8^). To remove IVs with chained imbalances, we applied a linkage disequilibrium (LD) factor (*R*^2^) of 0.001 within a clumping window width of 10 Mb [26]. If there was an LD effect among SNPs, then the SNP with the lowest *P* value was retained. In addition, we performed and calculated the *F* statistic to assess whether the retained SNPs may suffer from weak instrument bias, those SNPs with an *F*-statistic <10 weak instruments and excluded from the MR analysis [27].

### Statistical analyses

An MR analysis was performed utilizing frailty index-related SNPs as IVs to evaluate the association of frailty index with the common types of arthritis risk by utilizing the following statistical methods, including inverse-variance weighting (IVW), MR-Egger, weighted median (WM), weighted mode, and single-mode methods. Among them, the IVW method was the primary analysis method if all IV hypotheses were valid, and the other four additional sensitivity analysis methods were used to examine the robustness of the results [28]. The odds ratios (ORs) and 95% confidence intervals (CIs) for the risk of common arthritis were calculated.

To assess the validity and reliability of the resulting data, we performed sensitivity analysis, including heterogeneity tests, horizontal multiple validity tests, single SNP analysis, and leave-one-out analysis. Firstly, Cochrane’s *Q* test was used to evaluate heterogeneity among IVs in the fixed-effect IVW method. If the *P* value of Cochrane’s *Q* test <0.05, then we proceeded with the IVW method under multiplicative random effect to further estimate the effect. Moreover, we employed the MR-Egger regression and MR pleiotropy residual sum and outlier (MR-PRESSO) methods to identify potential uncorrelated horizontal pleiotropy. A *P*_intercept_ < 0.05 suggests there is directional pleiotropy among IVs. In MR-PRESSO analysis, heterogeneity, and horizontal pleiotropy in causal effect estimates were reduced by finding and removing outliers (SNP with a *P* value <1). Subsequently, the causal estimates were reassessed. In addition, the leave-one-out method was performed to ensure the stability of the results [29].

All analyses were performed using R packages MRPRESSO (version 1.0) and TwoSampleMR package (version 0.5.6), along with Mendelian randomization (version 0.5.0) in the R software (version 4.1.2). A value of *P* < 0.05 was considered statistically significant in the MR analysis.

## Results

### The causal effects of frailty index on common types of arthritis estimated by Mendelian randomization

The associations between genetic variants for frailty index and the common types of arthritis are summarized in Table 1. The *F*-value for all IVs was greater than 10, indicating there was no bias of weak IVs in the present study (Supplementary Tables S2–S3). With frailty index as an exposure factor and OA as an outcome variable, the results from the UK Biobank revealed that 15 valid SNPs were extracted, showing an estimated OR_IVW_ of 1.03 (95% CI: 1.01–1.05, *P* = 0.007) in the primary IVW method. In the FinnGen biobank database, the relationship between frailty index and OA was found to be more significant, with an OR_IVW_ of 1.55 (95%: 1.16–2.07, *P* = 0.003).

When RA as the outcome variable, the analysis results from the UK Biobank suggested frailty index significantly contributed to an increased risk of RA (OR_IVW_ = 1.03, 95% CI: 1.01–1.05, *P* < 0.001), and the results from the FinnGen biobank database showed that frailty index was significantly associated with a 4.57-fold risk of RA (95% CI: 1.35–96.49, *P* = 0.025). Furthermore, the frailty index was significantly associated with a 4.22-fold risk of PSA (95% CI: 1.21–14.67, *P* = 0.023) in the FinnGen biobank database. However, the analysis results from the UK biobank did not support the causal association between the frailty index and the risk of PSA. Additionally, no association was found between the frailty index and AS in either the UK Biobank or FinnGen Biobank databases (*P* > 0.050). These results demonstrated that the frailty index was positively associated with the risk of OA, RA, and PSA, not AS.

### Sensitivity and pleiotropy analysis

We conducted sensitivity tests in this study (Table 2). Regarding OA, in the UK Biobank data, we found that the association of frailty index with OA risk showed no evidence of heterogeneity but significant directional pleiotropy, according to MR-Egger regression method (*P*_intercept_ < 0.05) and the MR-PRESSO global test (*P* < 0.001), We further found that rs1363103, rs2071207, rs9275160 was a potential outlier, and after omitting these three outliers, no significant heterogeneity and pleiotropy was found in the causal association of frailty index and OA. And the relationship between frailty index and OA (OR_adjusted IVW_ = 1.03, 95% CI: 1.01–1.05; *P* = 0.001) remained unchanged. While in the FinnGen biobank database, we observed that genetic variants related to frailty index with OA may exhibit pleiotropy but no heterogeneity was detected.

**Table 2:** Heterogeneity test and horizontal pleiotropy test

Exposure	Outcome	Source	Heterogeneity test			MR-Egger pleiotropy test		MR-PRESSO global test		MR-PRESSO distorted outlier test
			Method	*Q* (*P* value)	Adjusted *Q* (*P* value)	Intercept (*P* value)	Adjusted intercept (*P* value)	RSSobs (*P* value)	Adjusted RSSobs (*P* value)	Outlying SNPs
Frailty index	Osteoarthritis	UKB	MR Egger	21.18 (0.069)	10.10 (0.432)	2.54E−03 (0.014)	1.27E−03 (0.399)	41.32 (<0.001)	22.04 (0.121)	rs1363103, rs2071207, rs9275160
			IVW	34.24 (0.002)	10.88 (0.453)					
		FINN	MR Egger	41.89 (3.5E−05)	NA	0.01 (0.377)	NA	19.74 (0.14)	NA	NA
			IVW	41.83 (2.2E−05)	NA					
Frailty index	Rheumatoid arthritis	UKB	MR Egger	29.82 (5.00E−03)	5.95 (0.877)	−3.50E−03 (1.05E−06)	−1.28E−03 (0.034)	286.17 (<0.001)	14.08 (0.484)	rs3959554, rs9275160
			IVW	198.20 (1.28E−34)	11.79 (0.463)					
		FINN	MR Egger	40.20 (6.66E−05)	4.75 (0.856)	−0.36 (0.047)	−0.03 (0.062)	347.81 (<0.001)	5.91 (0.912)	rs1363103, rs4952693, rs9275160
			IVW	239.72 (8.69E−44)	4.96 (0.891)					
Frailty index	Psoriatic arthropathies	UKB	MR Egger	23.68 (0.022)	5.35 (0.803)	−0.16 (5.06E−04)	0.01 (0.871)	1074.63 (<0.001)	6.48 (0.856)	rs2396766, rs374943348, rs82334, rs9275160
			IVW	67.45 (2.36E−09)	5.88 (0.864)					
		FINN	MR Egger	14.89 (0.248)	NA	1.44 (0.023)	NA	26.50 (0.057)	NA	NA
			IVW	22.73 (0.045)	NA					
Frailty index	Ankylosing spondylitis	UKB	MR Egger	16.94 (0.077)	6.20 (0.720)	7.37E−04 (0.763)	4.25E−04 (0.205)	44.72 (<0.001)	10.02 (0.608)	rs9275160
			IVW	30.09 (0.002)	8.06 (0.623)					
		FINN	MR Egger	13.79 (0.314)	NA	−0.04 (0.426)	NA	16.77 (0.344)	NA	NA
			IVW	14.57 (0.335)	10.10 (0.432)					

We first did a raw MR analysis and got an uncorrected causal evaluation, and then, MR-PRESSO global and Outliers test was performed to find unstable SNPs (The *P* value of SNP < 1), and an adjusted MR analysis was performed again after removing them, and the heterogeneity and pleiotropy were re-evaluated

*FINN* FinnGen, *IVW* inverse variance weighted, *UKB* UK Biobank

With RA, both the Cochran’s Q (MR Egger) and Cochran’s Q _IVW *P* values for frailty index with RA were less than 0.05 in both UK Biobank and FinnGen Biobank databases, suggesting that genetic variants associated with frailty index and RA might exhibit heterogeneity. In addition, the MR-Egger regression indicated some level of horizontal pleiotropy (*P* < 0.05) in the analysis of the causal relationships between frailty index and RA also in both two Biobank databases. To address this, we used the MR-PRESSO method to eliminate outliers, two outliers (rs6959554, rs9275160) were removed from the UK Biobank, and three outliers (rs1363103, rs4952693, rs9275160) from the FinnGen Biobank in the association between frailty index and RA. After removing these outliers, no significant heterogeneity or pleiotropy was detected in the causal association between the frailty index and RA. The significant relationship between the frailty index and RA remained unchanged in both the UK Biobank (OR_adjusted IVW_ = 1.01, 95% CI: 1.00–1.02, *P* = 4.61E−04) and the FinnGen Biobank (OR_adjusted IVW_ = 2.29, 95% CI: 1.26–4.18, *P* = 0.007).

Regarding PSA, significant heterogeneity and pleiotropy were found in the association between the frailty index and PSA in the UK Biobank (*P* < 0.05). Four outliers (rs2396766, rs374943348, rs82334, rs9275160) were identified. However, even after removing these outliers, the relationship between the frailty index and PSA remained insignificant. Interestingly, in the FinnGen Biobank, there was no heterogeneity (*P* > 0.05) but pleiotropy (*P* < 0.05) in the association between the frailty index and PSA, and the MR-PRESSO global test indicated no obvious pleiotropy (*P* > 0.05).

For AS, no heterogeneity or horizontal pleiotropy was found in the association between the frailty index and AS in the FinnGen Biobank database. However, in the UK Biobank database, there was heterogeneity, horizontal pleiotropy, and one outlier (rs9275160) in the association between the frailty index and AS, and the relationship between the frailty index and AS remained insignificant.

**Fig. 2:**
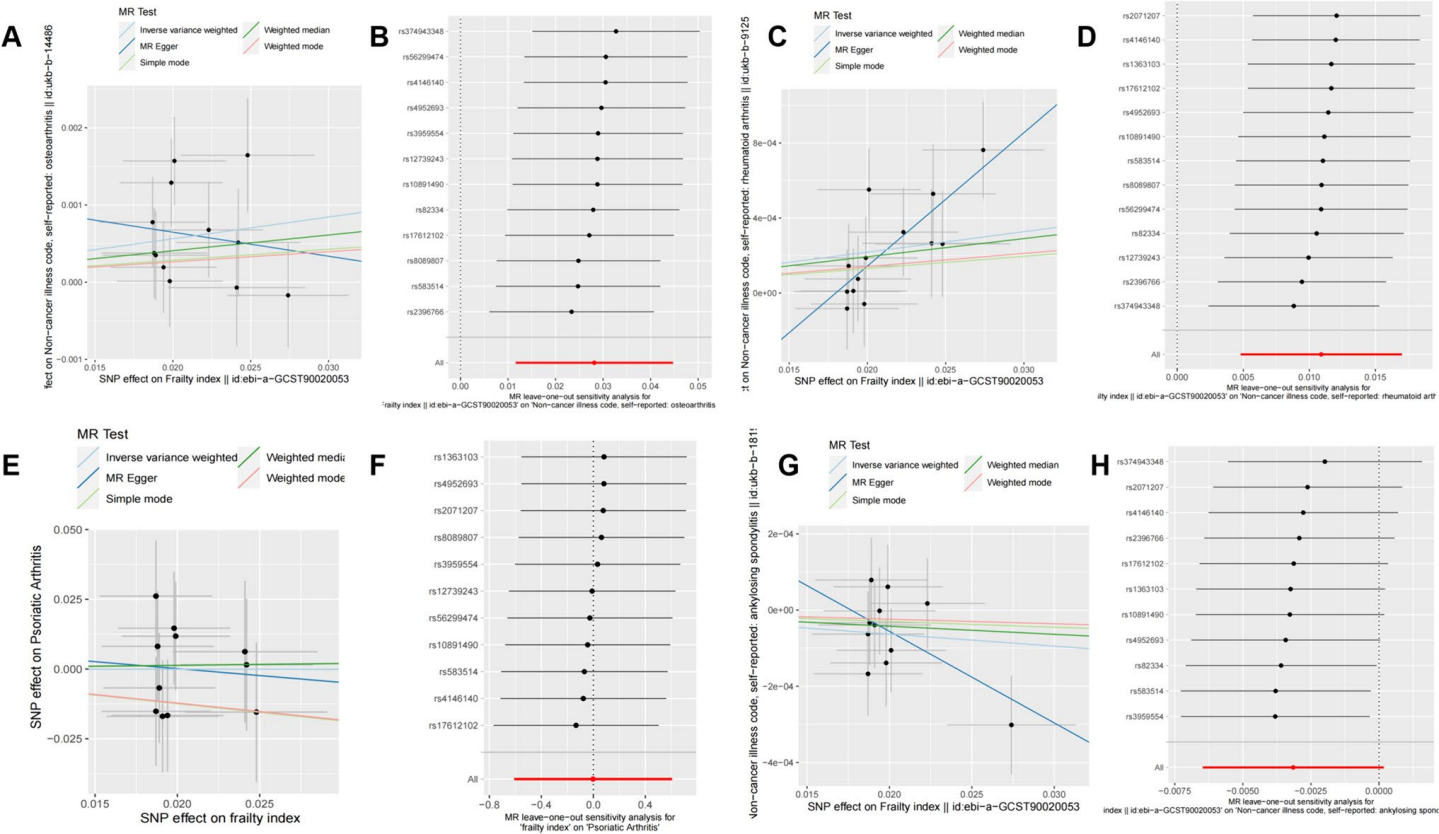
Visualization of the MR analysis of the effect of frailty index on common types of arthritis in the UK Biobank database. **A** Scatter plot of the primary MR analysis of the effect of frailty index on OA; **B** leave-one-out sensitivity analysis of the effect of frailty index on OA; **C** scatter plot of the primary MR analysis of the effect of frailty index on RA; **D** leave-one-out sensitivity analysis of the effect of frailty index on RA. **E** Scatter plot of the primary MR analysis of the effect of frailty index on PSA; **F** leave-one-out sensitivity analysis of the effect of frailty index on PSA. **G** Scatter plot of the primary MR analysis of the effect of frailty index on AS; **H** leave-one-out sensitivity analysis of the effect of frailty index on AS

**Fig. 3:**
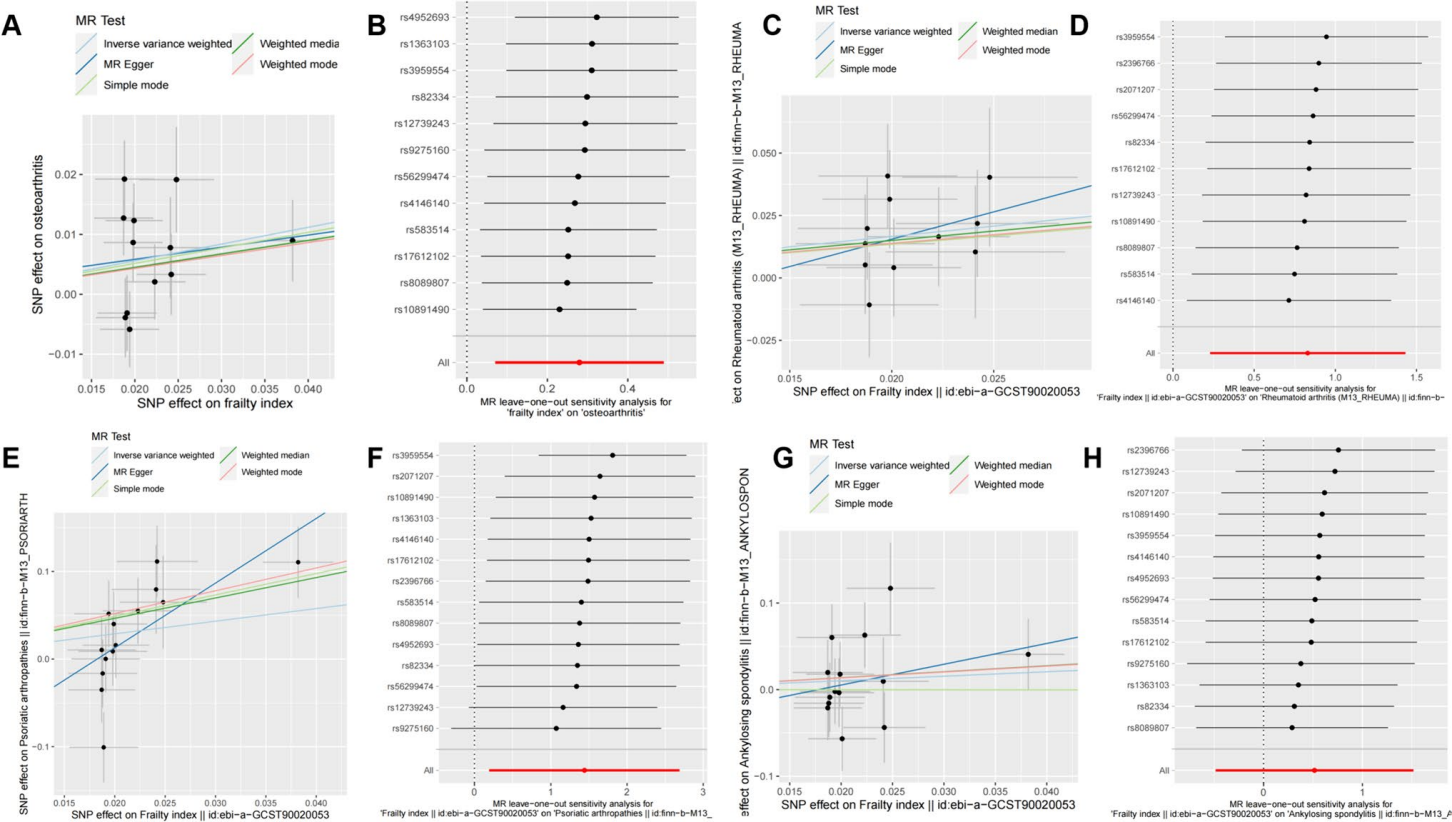
Visualization of the MR analysis of the effect of frailty index on common types of arthritis in the FinnGen Biobank database. **A** Scatter plot of the primary MR analysis of the effect of frailty index on OA; **B** leave-one-out sensitivity analysis of the effect of frailty index on OA; **C** scatter plot of the primary MR analysis of the effect of frailty index on RA; **D** leave-one-out sensitivity analysis of the effect of frailty index on RA. **E** Scatter plot of the primary MR analysis of the effect of frailty index on PSA; **F** leave-one-out sensitivity analysis of the effect of frailty index on PSA. **G** Scatter plot of the primary MR analysis of the effect of frailty index on AS; **H** leave-one-out sensitivity analysis of the effect of frailty index on AS

We also applied the leave-one-out method to test data heterogeneity, the results showed that there was no single SNP markedly altering the overall results in the association of frailty index with outcome OA, RA, and PSA (Figs. 2, 3).

## Discussion

In the current study, we applied a large-scale comprehensive MR analysis to investigate the potential causal association between frailty index and common types of arthritis in the UK Biobank and FinnGen Biobank databases. In our result, frailty was found to be likely a genetic causal factor associated with increased risk of RA, OA, and PSA, not but AS.

Some epidemiological studies have investigated the association between frailty and OA, RA, PSA, and AS. A study based on the National Health and Nutrition Examination Survey and Mendelian randomization study reported that there is a causal relationship between frailty index and the risk of hospitals diagnosed OA. Moreover, individuals with frailty might be at a higher risk for osteoarthritis, and frailty may be a better potential marker for OA than age though aging is known to be the strongest risk factor for OA [30]. Also, there is much evidence showing that individuals with OA have a higher prevalence and incidence of frailty than those without OA [14, 15, 31, 32]. Additionally, Terence W. O’Neill et.al has found that patients with OA who presented frailty had higher mortality following the total hip arthroplasty or total knee arthroplasty when compared with those who were non-frailty [33]. All of the above evidence indicated that both frailty and OA play important roles in each other’s development, which is similar to our MR results between the UK Biobank and FinnGen Biobank databases. In terms of RA, some studies suggest frailty is more prevalent in RA patients than those without RA [12, 34–36], and an MR study based on the IEU GWAS database reported that frailty may be a risk factor for RA [37], our results are in line with the above findings. Regarding PSA and AS, two prior studies demonstrated that frailty was common in RA and PSA, and the prevalence of frailty was almost half [16, 38]. However, there is no literature to investigate the effect of frailty on PSA and AS. Our findings showed that there is a strong association between frailty and the risk of PSA in the FinnGen Biobank database, but not in the UK Biobank database. The differences in the observed associations between the frailty index and PSA may be attributed to differences in sample size, population characteristics, and genetic heterogeneity between the two databases. However, frail individuals should remain vigilant that frailty is an important risk factor for PSA. In addition, we proposed more observational studies in the future that could examine the role of frailty in the development of PSA.

There are currently numerous speculations regarding the reasons behind the frailty as the cause of OA, RA, and PSA. Firstly, the levels of pro-inflammatory factors (such as IL-6, TNF-a, CRP) in the systemic circulation are elevated in a state of frailty [39]. The accumulation of these inflammatory cytokines may lead to a chronic inflammatory state, poor physical performance and decreased muscle strength in turn leads to increased burdens on the joints [40–42], which may increase the risk of OA, RA, and PSA. In addition to this, sarcopenia and frailty are extensively entwined, which can cause diminished activity levels in turn reduces the strength of the muscles around the joints and increases the risk of fall or arthroplasty, all of the above may increase the risk of developing arthritis [43]. However, the pathogenesis of RA, OA, and PSA were multifactorial, including genetics, immune response, and environmental factors, frailty may be considered as one of the potential factors contributing to the development and progression of those diseases. Thus, strengthening the prevention of frailty and providing long-term care management for frail patients is necessary.

Considering the presence of overlapping genetic architecture between the frailty index and four common types of arthritis, several approaches were utilized to investigate this relationship. Initially, the search results for phenotypes linked to SNPs associated with the frailty index in the GWAS Catalog revealed that these SNPs were solely connected to the frailty index and not arthritis. Furthermore, after removing the SNPs in the HLA gene region shared by frailty index and arthritis in sensitivity analysis [44], the observed relationship between frailty index and arthritis remained stable. Further studies are required to confirm the associations with OA, RA, and PSA are likely to be due to frailty.

There are several notable strengths of our study. Firstly, our MR study is the first to comprehensively evaluate the causal link between the frailty index with the common types of arthritis in two databases, and the MR analysis method enables us to account for potential confounding factors. In addition, we use multiple statistical methods for validation to improve the accuracy of the results, and we also perform sensitivity analysis to verify the stability and reliability of our findings. Despite the strengths, there are also several limitations in our study that warrant consideration. Firstly, all the participants in our study were the European descent, thus the results of this study should be carefully considered for generalizability to other ethnicities or geographic regions. Secondly, the diagnosis of OA and RA in this study was based on the self-report of the patient, which may bring about the misclassification bias. Last but not least, we used other robust MR methods to handle the horizontal pleiotropy, some robust MR methods were also used in this study. After removing all identified outliers, no significant heterogeneity was found in OA and RA. Higher-quality GWAS and MR analyses are thus needed to address these limitations in the future.

## Conclusion

In conclusion, there appears to be a causal relationship between frailty and RA, OA, and PSA, not but AS. Our results indicate that individuals with a history of frailty require specific clinical attention to prevent the development of RA, OA, and PSA, especially RA and PSA. Further studies are needed to examine the biological mechanisms underlying this association.

### Supplementary Information

Below is the link to the electronic supplementary material.

Supplementary file1 (XLSX 22 KB)

## Data Availability

No datasets were generated or analysed during the current study. All data reported in this paper will be shared by the lead contact upon request. This study did not report the original code.

## Declarations

### Inclusion and diversity

We support inclusive, diverse, and equitable conduct of research.
